# Gender differences in the prevalence and association between smartphone addiction and musculoskeletal disorders among vocational school students in Shanghai

**DOI:** 10.1016/j.pmedr.2025.103295

**Published:** 2025-11-01

**Authors:** Rongxuan Zhai, Xudong Wu, Tao Chen, Lejun Wang

**Affiliations:** a Sport and Health Research Center, Shanghai YangZhi Rehabilitation Hospital (Shanghai Sunshine Rehabilitation Center), Physical Education Department, Tongji University, Shanghai, China; b Shanghai Advanced Technical School, Shanghai University of Engineering Science, Shanghai, China

**Keywords:** Musculoskeletal disorders, Smartphone addiction, Vocational school students, Prevalence, Gender differences, Logistic regression

## Abstract

**Objectives:**

This study examined the prevalence of smartphone addiction and musculoskeletal disorders (MSDs), their association, and gender differences among vocational school students in Shanghai.

**Methods:**

A cross-sectional survey was conducted among 4897 students (61 % male) using the Mobile Phone Addiction Tendency Scale (MPATS) and the Standardized Nordic Questionnaire from March to April 2023. Binary logistic regression was applied to estimate adjusted odds ratios (ORs) for MSDs, including gender interaction terms.

**Results:**

Overall, 38.0 % of students reported MSDs (42.3 % among females vs. 35.2 % among males), most common in the neck (23.5 %), lower back (19.5 %), and shoulders (16.5 %), where females exhibited a higher prevalence at specific sites. Smartphone addiction was prevalent in 70.5 % of participants, significantly higher in females (76.5 %) than males (66.6 %). Both MPATS scores and addiction status were significantly associated with MSDs in the neck, shoulders, back, wrists, and elbows (*p* < 0.05), with the greatest risk noted for elbow pain (MPATS OR = 1.05, 95 %CI: 1.04, 1.06; addiction OR = 2.37, 95 %CI: 1.75, 3.20). Gender interactions were not significant.

**Conclusion:**

Smartphone addiction and MSDs are highly prevalent among vocational students, particularly females. The significant association between addiction and MSDs highlights the need for targeted interventions to reduce health risks linked to excessive smartphone use.

## Highlights

• A large sample of a vulnerable and underrepresented population.
• Quantified smartphone addiction and musculoskeletal disorders prevalence.
• Found gender differences in both conditions and strong links within each gender.

## 1 Introduction

Musculoskeletal disorders (MSDs) are a prevalent global health issue, with some studies reporting an incidence as high as 55 % in specific populations (Lucas et al., 2022). While much of the research focuses on adults, MSDs among adolescents also warrant significant attention due to their unique developmental and behavioral patterns. The prevalence of MSDs among adolescents is about 47.4 %, and 2.9 % of individuals under 24 sought medical care for MSDs (Navarro et al., 2024). MSDs in adolescents not only cause pain and poor posture but also negatively impact academic performance and quality of life (Figueiredo et al., 2024). Moreover, research indicates that women are more prone to osteoarthritis and joint pain than men, similar findings also exist in adolescent populations (Martins et al., 2020). Therefore, there is an urgent need to identify modifiable factors associated with the risk of MSDs and to examine potential gender differences in these risk factors among college students to inform early preventive strategies.

Existing systematic reviews suggest a positive association of electronic, mobile phone usage with MSDs (Young et al., 2022; Zirek et al., 2020). Recent studies across different populations, including those involving university students, have reported a significant association of smartphone addiction or excessive smartphone use with overall MSDs(Alghadir et al., 2025; Gholami et al., 2025; Hanphitakphong et al., 2021; Hassaan et al., 2024; Jacquier-Bret and Gorce, 2023; Lin et al., 2022; Mokhtarinia et al., 2024). Excessive smartphone use has also been associated with discomfort and pain in specific body regions, particularly the neck, shoulders, wrists/thumb, with poor posture, duration of use, and time of day identified as key contributing factors. For example, the reclining postures commonly adopted during evening smartphone use have been shown to increase the risk of musculoskeletal discomfort, while specific hand-held postures contribute to pain and discomfort in the thumb and wrist, particularly among right-handed students. Moreover, gender differences are evident in smartphone addiction patterns: women are more likely to develop social media addiction, while men are more prone to gaming addiction (Tu et al., 2023). It is unclear whether there is a gender difference in the association between smartphone addition and MSDs. The comparison of phone addiction between adolescent males and females has also been rarely studied.

China's vocational education system, comprising 7085 schools with 12.98 million enrolled students in 2023 (China, 2024). Vocational schools in China serve adolescents who transition from junior high school but encounter barriers to traditional high school education due to academic, socioeconomic, or personal circumstances. Research indicates that vocational students are more likely to be physically inactive and engage in sedentary screen-based activities (Hankonen et al., 2017) compared to their peers, heightening their susceptibility to compulsive smartphone use and addiction. Despite representing a significant population, vocational students are an understudied group in public health research. Socioeducational challenges, such as behavioral vulnerabilities (for example, limited self-regulation and disrupted familial support) (Zhang et al., 2023), may further increase their risk of excessive smartphone use and potential addiction. However, the relationship between smartphone addiction, MSDs, and gender disparities is not well understood in this demographic. To address this gap, we conducted a cross-sectional study involving 4897 vocational students in Shanghai (2992 males and 1905 females) using the Mobile Phone Addiction Tendency Scale (MPATS) and the Nordic Musculoskeletal Questionnaire. Our objectives were threefold: (1) to quantify the prevalence of smartphone addiction and MSDs, (2) to investigate their correlation, and (3) to analyze gender-specific patterns in these associations. By focusing on this overlooked demographic, our study enhances the understanding of how digital behavior intersects with physical health in marginalized youth while highlighting gender-related health disparities. The findings are particularly relevant for educators and policymakers, providing evidence to inform prevention programs that address both addiction and ergonomic practices. Additionally, by shedding light on the increased susceptibility to MSDs among females, this research supports the development of gender-responsive interventions aimed at reducing the long-term societal impacts of musculoskeletal morbidity.

## 2 Methods

### 2.1 Participants

From March to April 2023, a stratified random sampling approach was employed to ensure representative coverage of vocational schools across Shanghai. There are 40 vocational schools distributed across the eastern, southern, western, northern, and central districts of the city. In each district, 25 % of the schools were randomly selected, yielding a total of 10 schools included in the survey. Within each selected school, systematic sampling was then applied. All majors were randomly coded, and classes were chosen sequentially according to grade level (first, second, and third year), with four classes selected per grade. Questionnaires were subsequently distributed to all students enrolled in the selected classes. A total of 5020 questionnaires were distributed and all 5020 questionnaires were returned, with 4897 valid responses, resulting in an effective response rate of 97.4 %. Among the respondents, 2992 were male and 1905 were female.

### 2.2 Measures

Prior to the formal survey, a pilot test was conducted to assess the reliability of the questionnaire, yielding a Cronbach's α coefficient of 0.90, indicating excellent internal consistency. Trained surveyors entered classrooms to provide a standardized instructions on the study's purpose, objectives, and procedures. The content and structure of the questionnaires were explained in detail, including examples of how to complete items. Students were informed that participation was voluntary, responses would remain anonymous, and the data would be used exclusively for research purposes. Verbal explanations were supplemented with written instructions on the front page of the questionnaire. The survey took approximately 15 min to complete, during which surveyors remained present to clarify any questions and offer assistance as needed. After completion, surveyors reviewed the questionnaires for completeness before collecting them. The studies involving human participants were reviewed and approved by the Ethics Committee of Shanghai Yangzhi Rehabilitation Hospital (2020-074). The participants provided their written informed consent to participate in this study.

#### 2.2.1 Mobile phone addiction measures

Mobile phone addiction survey uses the mobile phone addiction tendency scale (MPATS), developed by Xiong Jie (Li et al., 2019; Jie et al., 2012). This scale has been widely validated in Chinese populations and has demonstrated good psychometric properties. It consists of 16 items across four dimensions: (1) withdrawal symptoms, (2) salience behaviors, (3) social comfort, and (4) mood changes. Each item is rated on a five-point Likert scale (1 = strongly disagree, 5 = strongly agree), yielding a total score ranging from 16 to 80. A cutoff score of 48 or higher indicates smartphone addiction, with higher scores reflecting more severe addiction. Previous studies have reported high internal consistency (Cronbach's α = 0.83) and excellent construct validity (Kaiser–Meyer–Olkin value = 0.91) (Li et al., 2019) for this scale.

#### 2.2.2 MSDs measures

MSDs were assessed using a modified version of the Standardized Nordic Musculoskeletal Questionnaire (NMQ) (Kuorinka et al., 1987), adapted to align with the objectives of this study. The final questionnaire assessed joint pain in five body regions: neck, shoulders, back, elbows, and wrists. It evaluated four types of symptoms, including soreness, numbness, pain, and restricted movement, over the past 12 months. MSDs were defined as the presence of any symptom lasting for more than 24 h without recovery after rest, excluding cases attributed to acute internal conditions, trauma, or other diseases. The original and modified versions of the NMQ have demonstrated high reliability, with Cronbach's α ranging from 0.85 to 1.00 in previous studies (Chairani, 2020; López-Aragón et al., 2017).

### 2.3 Statistical analysis

The prevalence of MSDs and mobile phone addiction was analyzed using frequencies (percentages), and chi-square tests were conducted to compare the differences in prevalence rates between genders. Mobile phone addiction scores were described using means ± standard deviations (SD), and independent samples *t*-tests were used to analyze gender differences. Binary logistic regression analysis was used to estimate were used to estimate adjusted odds ratios (ORs) and their 95 % confidence intervals (CIs) for associations between mobile phone addiction and MSDs stratified by gender and in the total sample. Forest plots were generated to visually display the effect sizes of the predictors. Interactions of gender with mobile phone addiction were also tested, to examine any potential modifying effects. A significance level of 0.05 was used for all tests. The data analysis was conducted using SPSS 29.0 (IBM Corp., Armonk, NY, USA).

## 3 Results

### 3.1 Comparison of MSDs between males and females

As shown in Table 1, the overall prevalence of MSDs among vocational school students in Shanghai was 38.0 % (1859 students). Female students exhibited a higher prevalence (42.3 %, 806 students) compared to male students (35.2 %, 1053 students). In terms of regional distribution, the neck had the highest prevalence of MSDs (23.5 %, 1152 students), followed by the lower back (19.5 %, 957 students) and the shoulders (16.5 %, 808 students). Female students reported higher rates of MSDs in the neck, shoulders, and lower back compared to their male counterparts. Specifically, the prevalence rates were 25.8 %, 21.0 %, and 23.8 %, respectively, for female students, while male students had rates of 22.1 %, 13.6 %, and 16.8 % (Table 1).

**Table 1:** Comparison of the prevalence of musculoskeletal disorders in various body parts among different genders among vocational school students in Shanghai in 2023.

	Male, n (%)	Female, n (%)	Total, n (%)	X^2^	P
Number	2992	1905	4897		
Neck	661 (22.1)	491 (25.8)	1152 (23.5)	8.8	<0.01
Shoulder	408 (13.6)	400 (21.0)	808 (16.5)	46.2	<0.01
Lower Back	503 (16.8)	454 (23.8)	957 (19.5)	36.5	<0.01
Wrist	201 (6.7)	122 (6.4)	323 (6.6)	0.2	0.67
Elbow	110 (3.7)	67 (3.5)	177 (3.6)	0.1	0.77
Total	1053 (35.2)	806 (42.3)	1859 (37.8)	25.0	<0.01

**Notes:** Musculoskeletal disorders were defined as the presence of soreness, numbness, pain, and restricted movement lasting more than 24 h without recovery after rest, excluding cases attributed to acute internal conditions, trauma, or other diseases.

### 3.2 Comparison of mobile phone addiction between males and females

As shown in Table 2, the overall detection rate of mobile phone addiction is 70.5 % (3450 cases). The detection rate among females (1457 cases, 76.5 %) is significantly higher than that among males (1993 cases, 66.6 %). The Mobile Phone Addiction Scale scores for females were significantly higher than those for males (Table 2).

**Table 2:** Mobile phone addiction cases and mobile phone addiction tendency scale score among different genders among vocational school students in Shanghai in 2023.

	Male	Female	Total	X^2^	P
Number	2992	1905	4897		
Mobile Phone Addiction Cases (%)	1993 (66.6 %)	1457 (76.5 %)	3450 (70.5 %)	54.1	<0.01
MPATS Score (Mean ± SD)	40.5 ± 12.3	42.2 ± 11.3	41.2 ± 11.9		<0.01

**Notes:** MPATS – Mobile Phone Addiction Tendency Scale. The MPATS total score ranges from 16 to 80. Scores of 48 or above are indicative of smartphone addiction, with higher scores representing more severe levels of addiction.

### 3.3 Analysis of the correlation between smartphone addiction and joint pain

**Fig. 1:**
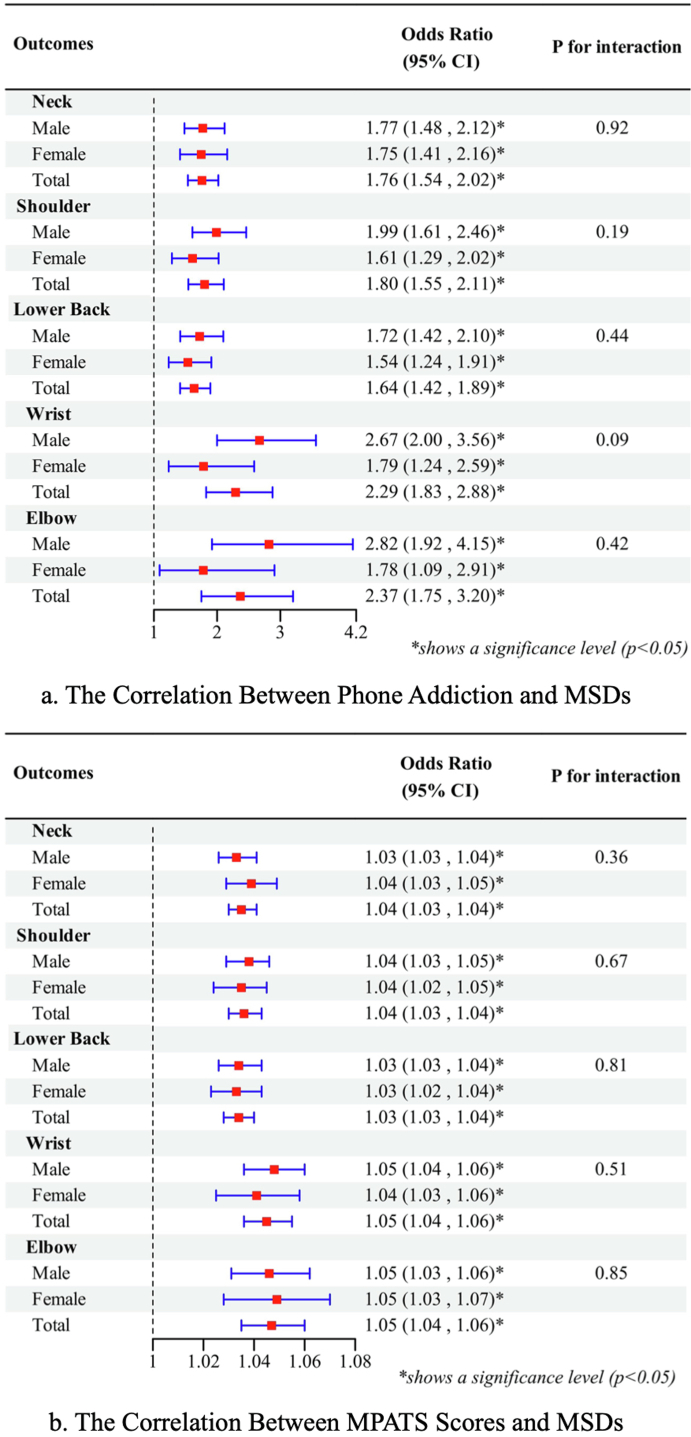
The correlation between smartphone addiction and musculoskeletal disorders among vocational school students in Shanghai in 2023. Notes: MSDs - Musculoskeletal disorders. MSDs were defined as the presence of soreness, numbness, pain, and restricted movement lasting for more than 24 hours without recovery after rest, excluding cases attributed to acute internal conditions, trauma, or other diseases. MPATS - Mobile Phone Addiction Tendency Scale. The MPATS total score ranges from 16 to 80. Scores of 48 or above are indicative of smartphone addiction, with higher scores representing more severe levels of addiction.

The associations between MPATS scores, mobile phone addiction (a binary variable: presence = 1, absence = 0), and MSDs across different body regions, with results stratified by gender and for the total population. As shown in Fig. 1, the forest plot illustrates the ORs and 95 % CIs for each predictor variable. Both MPATS scores and the presence of mobile phone addiction were significantly associated with pain in the neck, shoulders, back, wrists, and elbows (*p* < 0.05 for all). For neck pain, the odds ratios (OR) for MPATS scores were 1.03 (95 % CI: 1.03, 1.04) in males, 1.04 (95 % CI: 1.03, 1.05) in females, and 1.04 (95 % CI: 1.03, 1.04) overall. For mobile phone addiction, individuals with addiction had significantly higher odds of neck pain, with ORs of 1.77 (95 % CI: 1.48, 2.12) in males, 1.75 (95 % CI: 1.41, 2.16) in females, and 1.76 (95 % CI, 1.54, 2.02) overall. Similar trends were observed for shoulder pain, where MPATS scores showed ORs of 1.04, and mobile phone addiction was associated with ORs of 1.61–1.99. For back pain, MPATS scores had ORs of 1.03, while mobile phone addiction showed ORs of 1.54–1.72. The strongest associations for both MPATS scores and mobile phone addiction were observed with wrist pain, where MPATS scores showed an OR of 1.05 (95 % CI, 1.04, 1.06) and mobile phone addiction was associated with an OR of 2.29 (95 % CI, 1.83, 2.88) in the total population. Elbow pain also demonstrated significant associations, with MPATS scores showing an OR of 1.05 (95 % CI, 1.04, 1.06), and mobile phone addiction showing an OR of 2.37 (95 % CI, 1.75, 3.20). Across all analyses, gender interaction terms were not statistically significant (*p* > 0.05), indicating consistent associations between males and females (Fig. 1).

## 4 Discussion

This study is the first to investigate the prevalence of smartphone addiction and MSDs and to explore the correlations between these issues among a large sample of Chinese vocational school students. Our findings revealed that majority of the students (70.5 %) exhibited signs of smartphone addiction, while over one-third (38.0 %) reported experiencing MSDs. Notably, female students were found to be more likely than male students to suffer from both smartphone addiction and MSDs. Additionally, a significant association was identified between smartphone addiction and an increased prevalence of MSDs in both male and female students.

The prevalence of overall MSDs in our study (38.0 %) aligns with the systematic review reporting a 40 % prevalence of musculoskeletal pain among students aged 10–18, reinforcing the high burden of MSDs in younger populations (King et al., 2011). The neck emerged as the most frequently affected site, which is consistent with trends reported in a recent systematic review (Zirek et al., 2020). However, the prevalence of neck pain in our cohort (23.5 %) was lower than that observed in a general Iranian population during the COVID-19 period (43.5 % for neck discomfort, along with notable shoulder [35.1 %] and wrist [27.2 %] symptoms) (Mokhtarinia et al., 2024). Upper-limb symptoms were also prominent in other settings. Hand and wrist pain exceeded 25 % among Iranian college students, with right-hand thumb discomfort reaching 29.6 % (Banadaki et al., 2024). In the Saudi Arabian general population, 39.7 % of smartphone users reported wrist or thumb pain (Hassaan et al., 2024). Similarly, play-to-earn gamers exhibited markedly higher overall MSD prevalence (80 % over 12 months), with the upper back being the most frequently affected region (Gholami et al., 2025). These findings suggest that excessive and repetitive smartphone use may play a significant role in the development of MSDs. Additionally, neck (23.5 %) and lower back (19.5 %) pain rates in our cohort were lower than those among Chinese medical/dental students (44.4 % neck, 30.6 % lower back) and high school students in Shanghai (40.8 % neck, 33.1 % lower back) (Lin et al., 2022; Shan et al., 2013), potentially reflecting differences in academic demands, digital device usage intensity, or posture-related stressors. For instance, the Shanghai study explicitly linked pain prevalence to grade level and digital product use—a finding consistent with our focus on smartphone addiction as a potential risk factor (Shan et al., 2013). The higher MSD rates among female students in our study, particularly in the neck, shoulders, and lower back, mirror trends in prior studies, including the Shanghai cohort, and may stem from biological, postural, or behavioral disparities. Collectively, these findings underscore the pervasive nature of MSDs among youth, particularly in regions like the neck and lower back.

The smartphone addiction rate in our cohort (70.5 %) substantially exceeds rates reported in prior studies, such as 52.8 % among Chinese medical college students (Liu et al., 2022), underscoring the heightened vulnerability of vocational school populations. Our cohort revealed significantly higher addiction rates among female vocational students (76.5 % vs. 66.6 % in males), a finding consistent with studies from Saudi Arabia, which similarly reported greater vulnerability to smartphone addiction among females (Alghadir et al., 2025). This trend also aligns with observations in Chinese college students, where females exhibited heightened susceptibility to excessive smartphone use, potentially influenced by gender-specific engagement patterns, including stronger preferences for social interaction and reliance on smartphones for emotional coping (Li et al., 2020). These gender differences may also interact with physical health outcomes: prolonged smartphone use, particularly in females, may perpetuate poor postural habits (e.g., neck flexion during prolonged texting or scrolling), potentially amplifying MSDs risks in vulnerable regions like the neck and shoulders. Our results thus extend prior literature by contextualizing gender-specific vulnerabilities within an understudied population—vocational students—where socioeconomic, educational, and behavioral factors may intensify both addiction and its health consequences. Addressing these disparities requires interventions that account for gender-specific patterns of smartphone use, emotional regulation needs, and ergonomic education to mitigate MSD risks.

Our study demonstrates a significant positive association between smartphone addiction and MSDs, corroborating existing literature (Silva et al., 2016; Toh et al., 2017). Tapanya et al. (2021) found that prolonged smartphone use can lead to poor posture, causing long-term low-load muscle fatigue in the neck and shoulders, eventually resulting in discomfort or even MSDs in these areas. For students addicted to smartphones, this addiction typically manifests as extended periods of smartphone use, which in turn contributes to the development of MSDs. Similarly, Hanphitakphong et al. (2021) reported that prolonged smartphone use correlated with upper limb MSDs, consistent with our observations. These findings likely stem from repetitive hand movements, sustained muscle activation, and biomechanical stress during smartphone engagement, which increase demand on the neck and shoulders (Guan et al., 2016). Gorce and Jacquier-Bret (2023) further emphasized that that the time of day significantly influences postural load, with prolonged evening smartphone sessions exacerbating static muscle strain. Moreover, Gholami et al. (2025) found that smartphone gamers experienced greater prevalence of neck and upper back discomfort due to sustained flexed-head postures and repetitive hand movements. Physiological mechanisms, such as altered median nerve function, reduced pressure pain thresholds, and accelerated muscle fatigue, further amplify susceptibility to MSDs (İnal et al., 2015). While no interaction effects between gender and other risk factors emerged, gender disparities in both smartphone addiction (females: 76.5 % vs. males: 66.6 %) and MSD prevalence (e.g., neck, shoulders, lower back) suggest distinct risk profiles. Females' heightened vulnerability may reflect gendered patterns of smartphone use (e.g., social app engagement, prolonged texting) or psychosocial factors, such as elevated anxiety and stress levels, which are linked to compulsive device reliance (Gao et al., 2020; Graves et al., 2021). These disparities underscore the need for gender-tailored interventions addressing screen-time moderation and emotional regulation to mitigate MSD risks in vocational students. Our study addresses a significant gap by investigating smartphone addiction and MSDs among an underserved demographic: vocational school students. Additionally, we conduct an innovative exploration of gender disparities in this relationship.

However, several limitations must be acknowledged. First, our reliance on self-reported questionnaires for both MSD history and smartphone addiction introduces potential recall bias and social desirability bias, as participants may inaccurately recall past symptoms or underreport negative behaviors they perceive. Second, the cross-sectional design limits our ability to make causal inferences; longitudinal studies are necessary to clarify the temporal relationships between addiction and the development of MSDs. Third, unmeasured confounders, such as physical activity levels, ergonomic practices, and psychosocial stressors, may influence the observed associations.

## 5 Conclusion

This study found that both the rate of smartphone addiction and the prevalence of MSDs are relatively high among vocational school students in Shanghai, with more females affected than males. The link between smartphone addiction and MSDs shows the urgent need for targeted help to reduce the risks of using devices for long periods. Public health strategies should focus on vocational students and include programs that promote proper device use and limiting screen time.

## Availability of data and materials

The datasets used and/or analyzed during the current study are available from the corresponding author on reasonable request.

## CRediT authorship contribution statement

**Rongxuan Zhai:** Writing – original draft, Formal analysis. **Xudong Wu:** Methodology, Investigation, Data curation. **Tao Chen:** Writing – review & editing, Supervision. **Lejun Wang:** Writing – review & editing, Supervision, Formal analysis.

## Ethics approval and consent to participate

The studies involving human participants were reviewed and approved by the Ethics Committee of Shanghai Yangzhi Rehabilitation Hospital (2020-074), and was performed in accordance with ethical standards laid down in the 1964 Declaration of Helsinki and its later amendments. Written informed consent to participate in this study was provided by the participants. Informed consent to participate in the survey of the current study was obtained from the parents or legal guardians of any participant under the age of 16.

## Declaration of competing interest

The authors declare that they have no known competing financial interests or personal relationships that could have appeared to influence the work reported in this paper.

## Data Availability

Data will be made available on request.

## References

[bb0005] Alghadir A.H., Gabr S.A., Rizk A.A., Alghadir T., Alghadir F., Iqbal A. (2025). Smartphone addiction and musculoskeletal associated disorders in university students: biomechanical measures and questionnaire survey analysis. Eur. J. Med. Res..

[bb0010] Banadaki F.D., Rahimian B., Moraveji F., Varmazyar S. (2024). The impact of smartphone use duration and posture on the prevalence of hand pain among college students. BMC Musculoskelet. Disord..

[bb0015] Chairani A. (2020).

[bb0020] China, M. o. E. o. t. P. s. R. o (2024). Basic Information on the Development of National Education in 2023. http://www.moe.gov.cn/fbh/live/2024/55831/sfcl/202403/t20240301_1117517.html.

[bb0025] Figueiredo C.G., Santos V.S., Madureira E.V., Antunes J.S., do Espirito Santo C., Leite M.N., Yamato T.P. (2024). Most physical interventions for musculoskeletal pain in children and adolescents cannot be reproduced in clinical practice: a meta-research study of randomized clinical trials. BMC Musculoskelet. Disord..

[bb0030] Gao W., Ping S., Liu X. (2020). Gender differences in depression, anxiety, and stress among college students: A longitudinal study from China. J. Affect. Disord..

[bb0035] Gholami M., Ahmadi Shoar A., Kalantari R. (2025). Musculoskeletal disorders in smartphone play to earn gamers: A comparative study. J. Back Musculoskelet. Rehabil..

[bb0040] Gorce P., Jacquier-Bret J. (2023). Postural prevalence, time of day and spent time activities during smartphone weekday use among students: a survey to prevent musculoskeletal disorders. Heliyon.

[bb0045] Graves B.S., Hall M.E., Dias-Karch C., Haischer M.H., Apter C. (2021). Gender differences in perceived stress and coping among college students. PloS One.

[bb0050] Guan X.F., Fan G.X., Chen Z.Q., Zeng Y., Zhang H.L., Hu A.A., Gu G.F., Wu X.B., Gu X., He S.S. (2016). Gender difference in mobile phone use and the impact of digital device exposure on neck posture. Ergonomics.

[bb0055] Hankonen N., Heino M.T., Kujala E., Hynynen S.T., Absetz P., Araújo-Soares V., Borodulin K., Haukkala A. (2017). What explains the socioeconomic status gap in activity? Educational differences in determinants of physical activity and screentime. BMC Public Health.

[bb0060] Hanphitakphong P., Keeratisiroj O., Thawinchai N. (2021). Smartphone addiction and its association with upper body musculoskeletal symptoms among university students classified by age and gender. J. Phys. Ther. Sci..

[bb0065] Hassaan M.M., Jareebi M.A., AlKaabi H.A., Hobani A.H., Alfuhigi Y.M., Albahli N.K., Alrashed H., Alotaibi S.K., Almadi A.S., Iskander O.A., Alyahyawi K., Othman J.A., Borik W.S., Qaarie M.Y. (2024). Prevalence of thumb and wrist pain among smartphone users in the Saudi Arabian general population: a cross-sectional study. Cureus.

[bb0070] İnal E.E., Demİrcİ K., Çetİntürk A., Akgönül M., Savaş S. (2015). Effects of smartphone overuse on hand function, pinch strength, and the median nerve. Muscle Nerve.

[bb0075] Jacquier-Bret J., Gorce P. (2023). Effect of day time on smartphone use posture and related musculoskeletal disorders risk: a survey among university students. BMC Musculoskelet. Disord..

[bb0080] Jie Xiong, Zongkui Zhou, Wu Chen, Zhiqi You, Ziyan Z. (2012). Development of the mobile phone addiction tendency scale for college students. Chinese J. Mental Health.

[bb0085] King S., Chambers C.T., Huguet A., MacNevin R.C., McGrath P.J., Parker L., MacDonald A.J. (2011). The epidemiology of chronic pain in children and adolescents revisited: a systematic review. Pain.

[bb0090] Kuorinka I., Jonsson B., Kilbom A., Vinterberg H., Biering-Sørensen F., Andersson G., Jørgensen K. (1987). Standardised Nordic questionnaires for the analysis of musculoskeletal symptoms. Appl. Ergon..

[bb0095] Li G., Xie J., An L., Hou G., Jian H., Wang W. (2019). A generalizability analysis of the mobile phone addiction tendency scale for Chinese college students. Front. Psych..

[bb0100] Li L., Lok G.K.I., Mei S.L., Cui X.L., Li L., Ng C.H., Ungvari G.S., Zhang J., An F.R., Xiang Y.T. (2020). The severity of mobile phone addiction and its relationship with quality of life in Chinese university students. PeerJ.

[bb0105] Lin Y., Zhang X., Li H., Huang Y., Zhang W., Zhang C. (2022). Musculoskeletal pain is prevalent in Chinese medical and dental students: a cross-sectional study. Front. Public Health.

[bb0110] Liu H., Zhou Z., Huang L., Zhu E., Yu L., Zhang M. (2022). Prevalence of smartphone addiction and its effects on subhealth and insomnia: a cross-sectional study among medical students. BMC Psychiatry.

[bb0115] López-Aragón L., López-Liria R., Callejón-Ferre A.J., Gómez-Galán M. (2017). Applications of the standardized Nordic questionnaire: a review. Sustainability.

[bb0120] Lucas J., van Doorn P., Hegedus E., Lewis J., van der Windt D. (2022). A systematic review of the global prevalence and incidence of shoulder pain. BMC Musculoskelet. Disord..

[bb0125] Martins R.L., Carvalho N., Albuquerque C., Andrade A., Martins C., Campos S., Batista S., Dinis A.I. (2020). Musculoskeletal disorders in adolescents: a study on prevalence and determining factors. Acta Paulista De Enfermagem.

[bb0130] Mokhtarinia H.R., Torkamani M.H., Farmani N., Gabel C.P. (2024). Smartphone addiction prevalence, patterns of use, and experienced musculoskeletal discomfort during the COVID-19 pandemic in a general Iranian population. BMC Public Health.

[bb0135] Navarro G., Gómez-Autet M., Morales P., Rebassa J.B., Llinas Del Torrent C., Jagerovic N., Pardo L., Franco R. (2024). Homodimerization of CB(2) cannabinoid receptor triggered by a bivalent ligand enhances cellular signaling. Pharmacol. Res..

[bb0140] Shan Z., Deng G., Li J., Li Y., Zhang Y., Zhao Q. (2013). Correlational analysis of neck/shoulder pain and low back pain with the use of digital products, physical activity and psychological status among adolescents in Shanghai. PloS One.

[bb0145] Silva G.R., Pitangui A.C., Xavier M.K., Correia-Júnior M.A., De Araújo R.C. (2016). Prevalence of musculoskeletal pain in adolescents and association with computer and videogame use. J. Pediatr. (Rio J).

[bb0150] Tapanya W., Neubert M.S., Puntumetakul R., Boucaut R. (2021). The effects of shoulder posture on neck and shoulder musculoskeletal loading and discomfort during smartphone usage. Int. J. Ind. Ergon..

[bb0155] Toh S.H., Coenen P., Howie E.K., Straker L.M. (2017). The associations of mobile touch screen device use with musculoskeletal symptoms and exposures: A systematic review. PloS One.

[bb0160] Tu W., Nie Y., Liu Q. (2023). Does the effect of stress on smartphone addiction vary depending on the gender and type of addiction?. Behav. Sci. (Basel).

[bb0165] Young J.L., Snell M.G., Robles O., Kelso J.E., Kammitsis A.M., Cloutier N.K., DeVries A., Hilton C. (2022). Effects of electronic usage on the musculoskeletal system in adolescents and young adults: a systematic review. J. Musculoskelet. Disord. Treat..

[bb0170] Zhang H., Lin Y., Yang Y., Zhang J. (2023). Effect of alexithymia on mobile phone addiction among vocational college students: the mediation of negative evaluation fear and gender regulation. China J. Health Psychol..

[bb0175] Zirek E., Mustafaoglu R., Yasac Z., Griffiths M.D. (2020). A systematic review of musculoskeletal complaints, symptoms, and pathologies related to mobile phone usage. Musculoskelet. Sci. Pract..

